# S100A9 Increases IL-6 and RANKL Expressions through MAPKs and STAT3 Signaling Pathways in Osteocyte-Like Cells

**DOI:** 10.1155/2020/7149408

**Published:** 2020-02-19

**Authors:** Ryosuke Takagi, Eijiro Sakamoto, Jun-ichi Kido, Yuji Inagaki, Yuka Hiroshima, Koji Naruishi, Hiromichi Yumoto

**Affiliations:** ^1^Department of Periodontology and Endodontology, Institute of Biomedical Sciences, Tokushima University Graduate School, Tokushima 770-8504, Japan; ^2^Department of Oral Microbiology, Institute of Biomedical Sciences, Tokushima University Graduate School, Tokushima 770-8504, Japan

## Abstract

**Objective:**

Calprotectin is a heterocomplex of S100A8 and S100A9 and is mainly secreted from neutrophils, monocytes, and chondrocytes in inflammatory condition. Calprotectin binds to RAGE and TLR4 and induces the expression of proinflammatory chemokines and cytokines in various cells. Periodontitis is a chronic inflammatory disease that leads to gingival inflammation and alveolar bone resorption. Calprotectin levels in gingival crevicular fluid of periodontitis patients are higher than healthy patients. In the present study, the effects of S100A8 and S100A9 on the expressions of proinflammatory cytokines and bone metabolism-related factors in mouse osteocyte-like cells (MLO-Y4-A2) were investigated.

**Design:**

MLO-Y4-A2 cells were treated with S100A8 and S100A9, and the expressions of RAGE, TLR4, RANKL, and several inflammatory cytokines were analyzed by PCR and Western blotting or ELISA methods. To investigate the intracellular signaling pathways, phosphorylation of MAPK and STAT3 was determined by Western blotting, and chemical specific inhibitors and siRNAs were used.

**Results:**

Expressions of IL-6 and RANKL were increased by treatment with S100A9 but not S100A8. However, both S100A8 and S100A9 did not change expression of IL-1*β*, IL-8, and TNF-*α*. Although RAGE and TLR4 expressions were not upregulated by S100A9 treatment, transfection of siRNA for RAGE and TLR4 significantly decreased IL-6 and RANKL expressions. In addition, S100A9 activated p38, ERK, and STAT3 signaling pathways, and inhibitors for these factors significantly decreased S100A9-induced IL-6 and RANKL expressions.

**Conclusions:**

These results indicated that S100A9 induces IL-6 and RANKL production via engagement with RAGE and TLR4 signalings in osteocytes and suggested that S100A9 may play important roles in the periodontal alveolar bone destruction.

## 1. Introduction

S100A8 and S100A9 proteins are subunits of the calcium-binding protein and are abundant in myeloid cells such as neutrophils, monocytes, and chondrocytes [1]. The heterocomplex of S100A8 and S100A9 has been known as calprotectin, which is detected in various tissues. A previous study reported that S100A8 and S100A9 were expressed in murine macrophages, osteoblasts, and osteoclasts [2]. Although serum calprotectin levels in normal conditions are very low, that levels in several inflammatory diseases including inflammatory bowel disease, rheumatoid arthritis (RA), chronic bronchitis, and periodontitis are increased [3, 4]. Calprotectin acts in a cytokine-like manner by binding to cell surface receptors: Toll-like receptor 4 (TLR4) and receptor of advanced glycation end-products (RAGE) [5], and induces the expression of various proinflammatory chemokines and cytokines, such as C-X-C motif ligand (CXCL)1, interleukin (IL)-6, IL-8, and tumor necrosis factor (TNF)-*α* [6, 7]. Therefore, it has been considered that calprotectin belongs to damage-associated molecular patterns (DAMPs) or alarmin by increasing the expression of inflammatory mediators in several inflammatory diseases.

Periodontitis is a chronic inflammatory disease leading to the destruction of periodontal tissues such as connective tissue, periodontal ligament, and alveolar bone [8]. The pathogenesis of chronic periodontal disease is associated with host inflammatory responses to periodontal pathogens forming biofilms in periodontal pockets, and many cytokines produced from various periodontal tissue cells by these immune reactions regulate the pathophysiological conditions [9]. Lipopolysaccharide (LPS) derived from *Porphyromonas gingivalis* (*Pg*), a major pathogenic component, affects bone metabolisms and aggravates periodontitis. Liu et al. have recently reported that bacterial LPS inhibits osteoblastic differentiation and alkaline phosphatase (ALP) activity by suppressing the expressions of osteocalcin, Runx2, and ALP, and then downregulates bone matrix mineralization [10]. Moreover, LPS induces inflammatory cytokine levels, such as IL-1*β*, IL-6, IL-8, TNF-*α*, and S100A8/S100A9 from various cells in periodontal tissues [11–13]. In a recent study, high levels of calprotectin were detected in gingival crevicular fluid (GCF) from patients with periodontitis, and high levels of S100A8 and S100A9 were also detected in blood vessels in *Pg*-infected mice [14, 15]. These reports suggest that calprotectin expression levels in patients with periodontitis were increased by its causal agents, such as *Pg* LPS, and plays important roles in periodontal inflammatory responses.

Bone remodeling is maintained by osteoblasts, osteocytes, and osteoclasts, which are regulated by inflammatory factors and hormones [16]. Osteocytes constitute the main cellular component of mammalian bone, and represent more than 90% of all the bone cells [17]. Osteocytes regulate osteoblast and osteoclast activity and have been found to act as a key factor of bone remodeling and metabolisms by expressions of several pro- and antiosteoclastogenic factors such as high mobility group box 1 (HMGB1), receptor activator of nuclear factor-kappa B ligand (RANKL), macrophage-colony stimulating factor (M-CSF), and osteoprotegerin (OPG) [16, 18, 19]. RANKL encoded by tumor necrosis factor superfamily 11 (TNFSF11) gene is mainly expressed on the surface of osteoblasts and osteocytes. It has been reported that two receptors for RANKL are the membrane bound receptor, RANK, and soluble decoy receptor OPG, and RANK-RANKL signaling has important roles in activation of osteoclasts [20]. RANKL expression was upregulated in several chronic inflammatory diseases such as rheumatoid disease, ankylosing spondylitis, inflammatory bowel disease, and periodontitis [21]. Recently, osteocytes were shown as one source of RANKL and play an important role in osteoclast formation [16]. The upregulated RANKL levels are related to the number of *Pg* in clinically obtained periodontal tissues and LPS enhances RANKL expression in mouse osteoblasts and osteocytes [22, 23]. Moreover, periodontal therapy decreased serum RANKL levels in patients with periodontitis [24]. However, few studies have reported on the effects of calprotectin in bone metabolisms. Grevers LC et al. reported that bone destruction and active osteoclast number were decreased in S100A9 knockout mice with antigen-induced arthritis. On the other hand, S100A8 stimulation increased tartrate resistant acid phosphatase (TRAP) positive cells in mouse bone marrow cells [25]. Although these findings suggested that S100A8 and S100A9 have important roles in bone metabolisms, those mechanisms are not still clear.

In the present study, we focused on the effects of S100A8 and S100A9 on expressions of proinflammatory- and bone metabolism-related factors in osteocytes to elucidate mechanisms in aggravation of periodontitis.

## 2. Materials and Methods

### 2.1. Cell Culture

Mouse osteocyte-like MLO-Y4-A2 cells were kindly provided by Prof. T Sugimoto (Shimane University) with the consent of Prof. Lynda F Bonewald (Indiana University). MLO-Y4-A2 cells were cultured in *α*-minimum essential medium (*α*-MEM) containing 10% Fetal bovine serum (FBS) and antibiotics (100 U/mL penicillin G and 50 *μ*g/mL streptomycin: Meiji, Tokyo, Japan) on type I collagen-coated dish and plates (IWAKI, Shizuoka, Japan). The cells were maintained at 37°C in humidified atmosphere with 5% CO_2_ and the medium was changed every 2 days. On day 5 when the cells reached subconfluence, they were stimulated with 10–50 nM recombinant S100A8 (ATGen, Sampyeongdong, South Korea), 10–50 nM recombinant S100A9 (ATGen), or 500 ng/mL *Pg* LPS (Invivogen, San Diego, USA).

### 2.2. Determination of Cell Viability

Cell viability was determined using Cell Counting Kit-8 (CCK-8, Dojindo, Kumamoto, Japan). Osteocytes were seeded in type I collagen-coated 96-well plates at 5,000 cells/cm^2^ and precultured for 24 hours. After preculture, cells were stimulated with S100A8 (10–50 nM), S100A9 (10–50 nM), or *Pg* LPS (500 ng/mL) for 48 hours and incubated with CCK-8 solution for 4 hours. The absorbance of culture medium at 450 nm was measured using a microplate reader (iMark™ Microplate Reader, Bio-Rad, Hercules, CA, USA) and cell viability was calculated as a percentage compared to 100% of unstimulated control.

### 2.3. RNA Isolation and Polymerase Chain Reaction (PCR)

Total RNA was isolated from osteocytes using RNA iso plus (Takara Bio, Shiga, Japan), containing 38% phenol and chloroform according to the manufacturer's instructions, and its concentration and purity were analyzed using Nano Drop ND-1000 (Thermo Fisher Scientific, Waltham, MA, USA). First-strand cDNA was synthesized using PrimeScript II 1^st^ strand cDNA Synthesis Kit (Takara Bio) from 1 *μ*g isolated total RNA. Reverse transcription-PCR (RT-PCR) was performed using the TaKaRa PCR Thermal Cycler Dice (Takara Bio) and PCR products separated on 1.5% agarose gels with 0.5 *μ*g/mL ethidium bromide were visualized under ultraviolet light. Quantitative real-time PCR (qRT-PCR) was also performed using the CFX96 Touch Real-Time PCR Detection System (Bio-Rad) with SsoAdvanced Universal SYBER Green Supermix (Bio-Rad). All expression levels were normalized against the glyceraldehyde-3-phosphate dehydrogenase (GAPDH) housekeeping gene and calculated by 2^−∆∆Ct^ method. The sequences of the primers used for PCR are shown in Table 1.

### 2.4. Enzyme-Linked Immunosorbent Assay (ELISA)

After osteocytes were treated with S100A8 (10–50 nM), S100A9 (10–50 nM), or *Pg* LPS (500 ng/mL) for 48 hours, and the concentrations of IL-6 in cell culture medium and RANKL in whole-cell lysates were measured using ELISA kit (R&D systems, Minneapolis, MN, USA) according to the manufacturer's instruction.

### 2.5. Protein Extraction and Western Blot Analysis

Osteocytes cultured with S100A9 (50 nM) for 0.5–2 hours were collected using RIPA lysis buffer containing protease inhibitors cocktail and phosphatase inhibitor (Santa Cruz, Dallas, Texas, USA) and then homogenized using syringe and 23G needle. After centrifugation at 10,000 rpm for 10 min, the supernatant was collected as a cell lysate sample. Total protein content was measured using the protein assay BCA kit (Wako, Osaka, Japan) according to the manufacturer's instructions. Samples containing 30 *μ*g protein were separated on 10% sodium dodecyl sulfate-polyacrylamide gel electrophoresis (SDS-PAGE) and transferred to polyvinylidene difluoride (PVDF) membrane (Merck Millipore, Germany). Nonspecific protein binding was blocked using PVDF Blocking Reagent for Can Get Signal (Toyobo, Osaka, Japan) for 1 hour at room temperature and the membrane was incubated at 4°C for overnight with specific first-antibodies against anti-p38 (1/500 dilution, Cell Signaling Technology; CST, Boston, MA, USA), anti-phospho-p38(1/500 dilution, CST), anti-c-Jun N-terminal kinase (JNK) (1/1,000 dilution, CST), anti-phospho-JNK (1/1,000 dilution, CST), anti-extracellular signal-regulated kinase (ERK) (1/1,000 dilution, CST), anti-phospho-ERK (1/1,000 dilution, CST), anti-signal transducer and activator of transcription (STAT) 3 (1/500 dilution, BioLegend, San Diego, USA), anti-phospho-STAT3 (1/500 dilution, BioLegend), or anti-*β*-actin (1/10,000 dilution, CST). On the following day, the membranes were washed three times with tris-buffered saline buffer containing 0.05% Tween 20 (TBS/T) and incubated with horseradish peroxidase (HRP)-conjugated secondary antibodies (1/2,000 dilution, CST) for 1 hour at room temperature. Reactivity was visualized using ECL Western Blotting Detection Reagents (GE Healthcare Japan, Tokyo, Japan) and Image Quant LAS 500 (GE Healthcare) and analyzed using NIH image v.1.63 software (National Institutes of Health, USA). Protein levels were normalized to *β*-actin.

### 2.6. siRNA Transfection

When osteocytes reached 70% confluency, specific siRNA for RAGE (FlexiTube GeneSolution GS11596 for Ager, 50 nM, QIAGEN, Hilden, Germany), TLR4 (FlexiTube GeneSolution GS21898 for tlr4, 10 nM, QIAGEN) or negative control (Control siRNA-A: sc-37007, 10 nM or 50 nM, Santa Cruz) was transfected using Lipofectamin^TM^ RNAiMAX Transfection Reagent (Thermo Fisher Scientific) for 24 hours. After transfection with siRNA, cells were treated with S100A9 (50 nM) for 24–48 hours and the expression levels of IL-6 and RANKL were analyzed by qRT-PCR and ELISA.

### 2.7. Mitogen-Activated Protein Kinase (MAPK) and STAT3 Inhibitions

Osteocytes were pretreated with MAPK inhibitor including SB203580 (20 *μ*M) as p38 inhibitor, U0126 (10 *μ*M) as ERK inhibitor, SP600125 (10 *μ*M) as JNK inhibitor, or a STAT3 inhibitor, cryptotanshinone (10 *μ*M), for 1 h and then further treated with S100A9 (50 nM) for 24–48 hours. To determine the involvement of MAPK and STAT3 activations in IL-6 and RANKL productions in osteocytes, mRNA expressions and protein productions were analyzed by qRT-PCR and ELISA, respectively.

### 2.8. Statistical Analysis

Statistical analyses were performed by analysis of variance (ANOVA) and Tukey-Kramer methods. *P* value < 0.05 was considered statistically significant. All analyses were performed with Stat view v.5.0 (SAS Institute Inc. Cary, NC, USA).

## 3. Results

### 3.1. The Effect of S100A8, S100A9, and *Pg* LPS on Cell Viability

**Figure 1:**
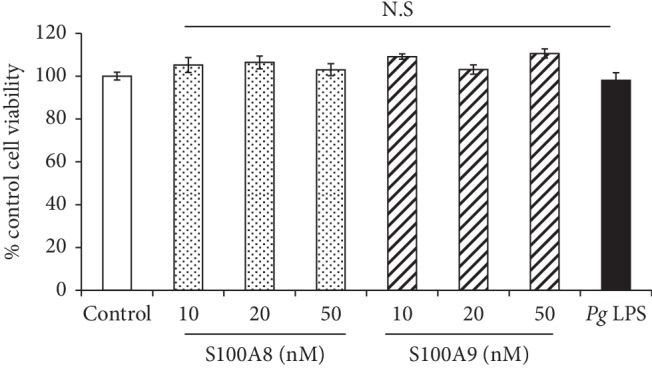
Effect of S100A8, S100A9, and *Pg* LPS on cell viability of osteocytes. When MLO-Y4-A2 cells reached subconfluence, cells were treated with S100A8 (10–50 nM), S100A9 (10–50 nM), and *Pg* LPS (500 ng/ml) for 48 hours, and cell viability was analyzed using CCK-8. Data are means ± SD and expressed as a percent of control (*n* = 5). N.S indicates no significant difference between the indicated groups and control group.

Osteocytes were treated with S100A8, S100A9 (10–50 nM), and *Pg* LPS (500 ng/mL) for 48 hours and analyzed cell viability. All tested concentrations of S100A8, S100A9, and *Pg* LPS did not affect the cell viability of osteocytes (Figure 1).

### 3.2. The Effect of S100A8 and S100A9 on mRNA Expressions of Proinflammatory Cytokines in Osteocytes

**Figure 2:**
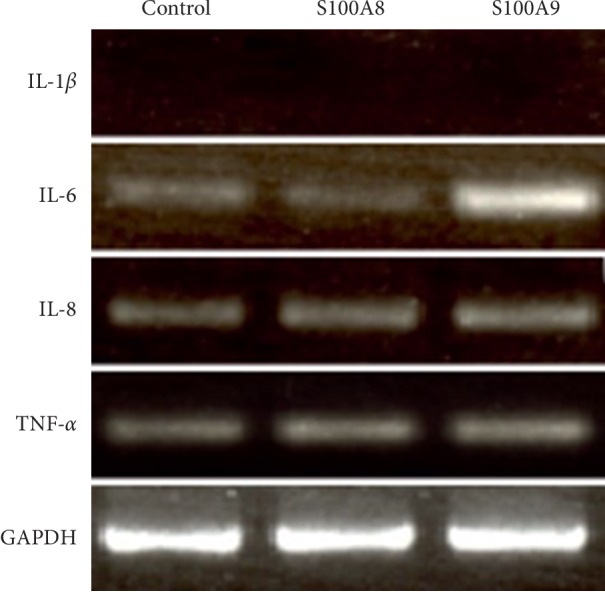
The effect of S100A8 and S100A9 on mRNA expressions of proinflammatory cytokines in osteocytes. MLO-Y4-A2 cells were cultured in *α*-MEM containing S100A8 (50 nM) or S100A9 (50 nM) for 24 hours, and mRNA expressions of IL-1*β*, IL-6, IL-8, andTNF-*α* were analyzed by RT-PCR.

The mRNA expressions of proinflammatory cytokines in S100A8 (50 nM)- or S100A9 (50 nM)-treated osteocytes were investigated by RT-PCR (Figure 2). IL-6 mRNA expression was upregulated by the stimulation with S100A9 but not S100A8. In contrast, neither S100A8 and S100A9 affected IL-8 and TNF-*α* mRNA expressions, and IL-1*β* mRNA was not detected in osteocytes.

### 3.3. Dose Effects of S100A9 on IL-6 and RANKL Expressions in Osteocytes

**Figure 3:**
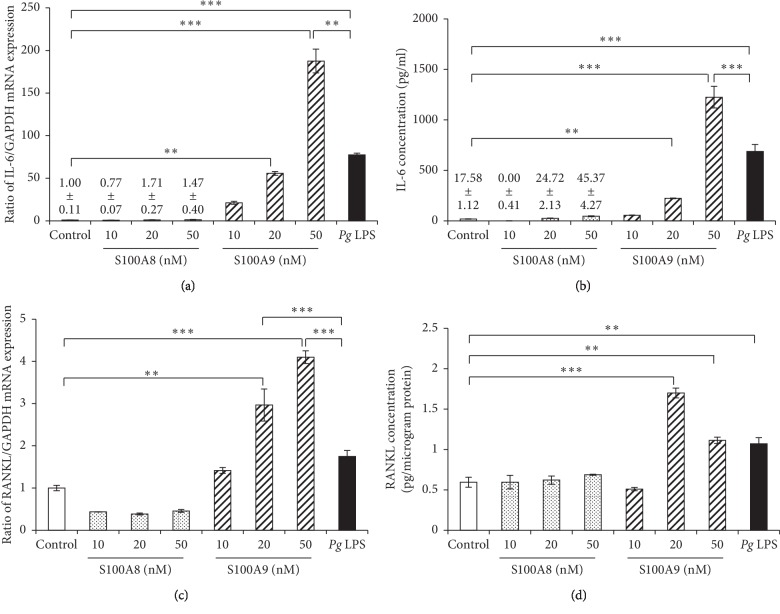
Dose effects of S100A8 and S100A9 on IL-6 and RANKL expressions in osteocytes. MLO-Y4-A2 cells were treated with S100A8 (10–50 nM), S100A9 (10–50 nM), and *Pg* LPS (500 ng/ml) for 24–48 hours. ((a) and (c)) After 24 hours of stimulation, IL-6 and RANKL mRNA expressions were analyzed by qRT-PCR. ((b) and (d)) IL-6 and RANKL production levels were measured using ELISA after treatment with a reagent for 48 hours. Data are means ± SD (*n* = 6). ^*∗∗*^*P* < 0.01 and ^*∗∗∗*^*P* < 0.001 show significant differences between the indicated groups.

S100A8 (10–50 nM) did not affect IL-6 mRNA expression and protein production in osteocytes up to 48 hours. The increasing tendency of IL-6 expression by the stimulation with S100A9 (10–50 nM) was observed, and the stimulation with S100A9 at 20 and 50 nM significantly increased both mRNA and protein levels of IL-6 (Figures 3(a) and 3(b)). Similarly, both mRNA and protein levels of RANKL were not changed by the stimulation with S100A8 (10–50 nM), but S100A9 significantly increased mRNA expression level of RANKL in a dose-dependent manner (Figure 3(c)). However, further increasing effect of S100A9 on RANKL protein production was observed at 20 nM compared with 50 nM (Figure 3(d)). *Pg* LPS was used as a positive control and significantly increased both IL-6 and RANKL mRNA expressions and protein productions in osteocytes. Interestingly, the increasing effects of S100A9 on both IL-6 and RANKL expressions were much stronger than *Pg* LPS.

### 3.4. The Effect of S100A9 on RAGE and TLR4 Expressions in Osteocytes

**Figure 4:**
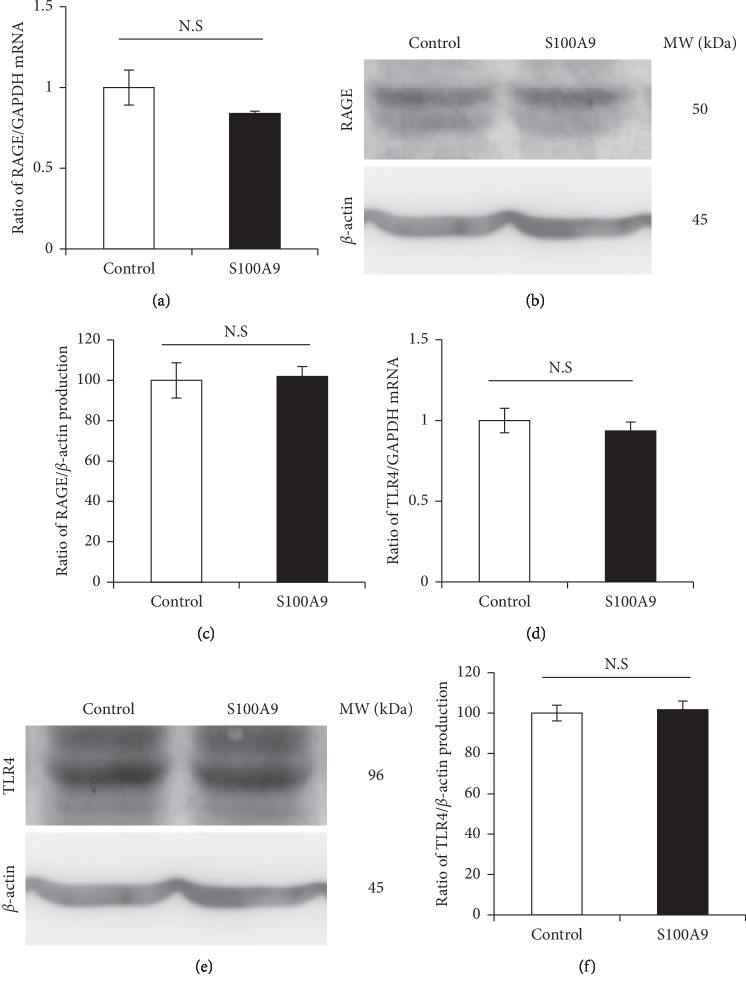
The effect of S100A9 on RAGE and TLR4 expressions in osteocytes. MLO-Y4-A2 cells were treated with S100A9 (50 nM) for 24–48 hours, and total RNA and cell lysate were isolated. ((a) and (d)) After 24 hours of stimulation with S100A9, RAGE and TLR4 mRNA expressions were analyzed by qRT-PCR. ((b) and (e)) RAGE and TLR4 protein productions after 48 hours of stimulation with S100A9 were analyzed by Western blotting. ((c) and (f)) RAGE and TLR4 protein production levels were analyzed by densitometric measurement. Data are means ± SD after normalizing to *β*-actin expression levels (*n* = 3). N.S indicates no significant difference between the indicated groups.

RAGE and TLR4 expressions in S100A9-treated osteocytes were analyzed by qRT- PCR and Western blotting. The stimulation with S100A9 did not affect both RAGE and TLR4 mRNA and protein levels (Figure 4).

### 3.5. The Effect of RAGE and TLR4 Knockdown on IL-6 and RANKL Expressions in Osteocytes

**Figure 5:**
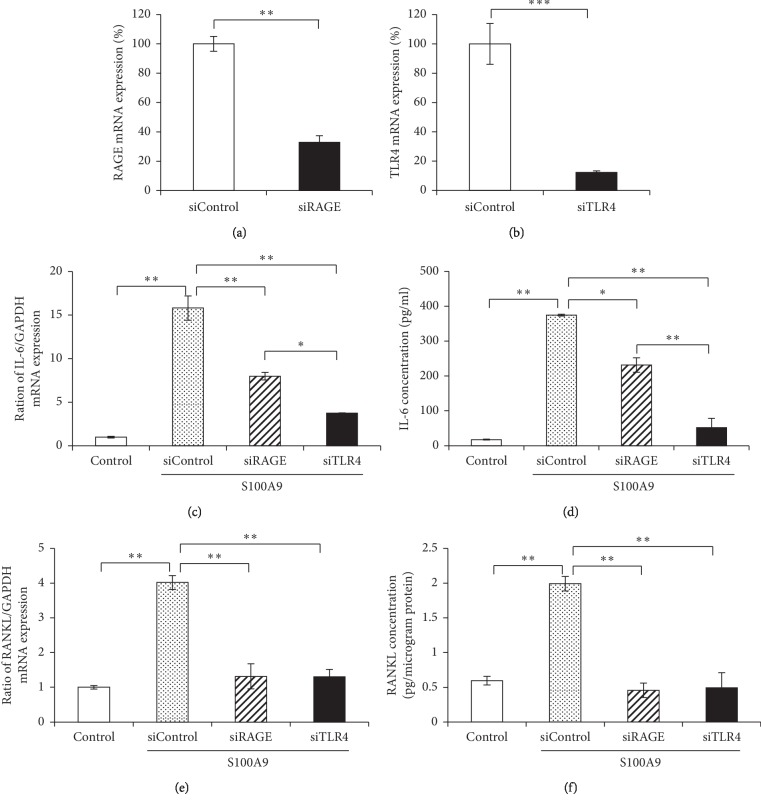
The effect of RAGE and TLR4 knockdown on IL-6 and RANKL expressions in osteocytes. When MLO-Y4-A2 cells reached 60–70% confluence, cells were transfected with RAGE-, TLR4-specific or negative control siRNA for 24 hours. ((a) and (b)) The effect of transfection with siRNA was determined by qRT-PCR. ((c) and (e)) Transfected cells were treated with S100A9 (50 nM) for 24 hours, and IL-6 and RANKL mRNA expressions were analyzed by qRT-PCR. ((d) and (f)) After 48 hours stimulation with S100A9 (50 nM), IL-6 and RANKL productions in transfected cells were measured using ELISA. All results of qRT-PCR were normalized by GAPDH mRNA expression levels. Data are means ± SD (*n* = 3). ^*∗*^*P* < 0.05 and ^*∗∗*^*P* < 0.01 show significant differences between the indicated groups.

To clarify the pathway of S100A9-induced IL-6 and RANKL expressions, osteocytes were transfected with siRAGE or siTLR4. Transfection with siRAGE or siTLR4 significantly inhibited RAGE and TLR4 mRNA expressions in osteocytes, respectively (Figures 5(a) and 5(b)). The knockdown of RAGE or TLR4 significantly decreased IL-6 mRNA and protein expressions in osteocytes, and siTLR4 transfection had a stronger inhibitory effect on IL-6 at both mRNA and protein levels compared with siRAGE transfection (Figures 5(c) and 5(d)). On the other hand, siRAGE and siTLR4 transfections significantly decreased RANKL at both mRNA and protein levels, but the significant difference between siRAGE and siTLR4 was not observed (Figures 5(e) and 5(f)).

### 3.6. The Effect of S100A9 on Phosphorylations of MAPK and STAT3

**Figure 6:**
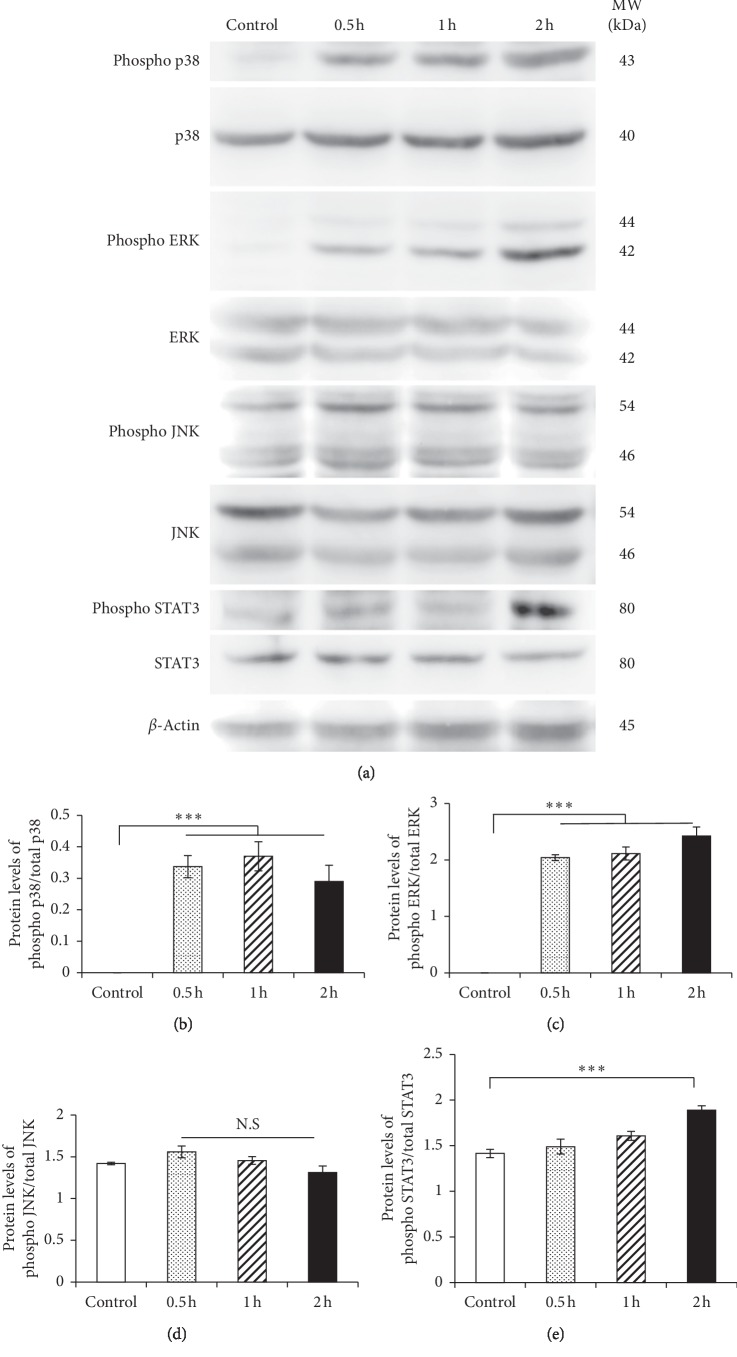
The effect of S100A9 on the phosphorylations of MAPKs and STAT3 in osteocytes. MLO-Y4-A2 cells were treated with S100A9 for 0.5–2 hours, and cell lysate from osteocytes was extracted. (a) Phosphorylations of MAPKs including p38, ERK, and JNK and STAT3 were determined by Western blotting. ((b)–(e)) Phosphorylation levels of MAPKs and STAT3 were analyzed by densitometric measurement. Data were normalized by nonphosphorylated total factors. Data are means ± SD (*n* = 3). N.S indicates no significant difference between the indicated groups and control group.

The phosphorylations of MAPKs and STAT3 in osteocytes treated with 50 nM S100A9 for 0.5–2 hours were analyzed by Western blotting. The increases of p38 and ERK phosphorylations were observed within 0.5–2 hours after the stimulation with S100A9 (Figures 6(a)–6(c)), but JNK phosphorylation level was not changed by the stimulation with S100A9 (Figures 6(a) and 6(d)). Moreover, the phosphorylation of STAT3 was significantly increased at 2 hours after S100A9 treatment, but this was not clearly observed at 0.5–1 hours (Figures 6(a) and 6(e)).

### 3.7. The Effect of MAPKs and STAT3 Inhibitions on IL-6 and RANKL Expressions in S100A9-Stimulated Osteocytes

**Figure 7:**
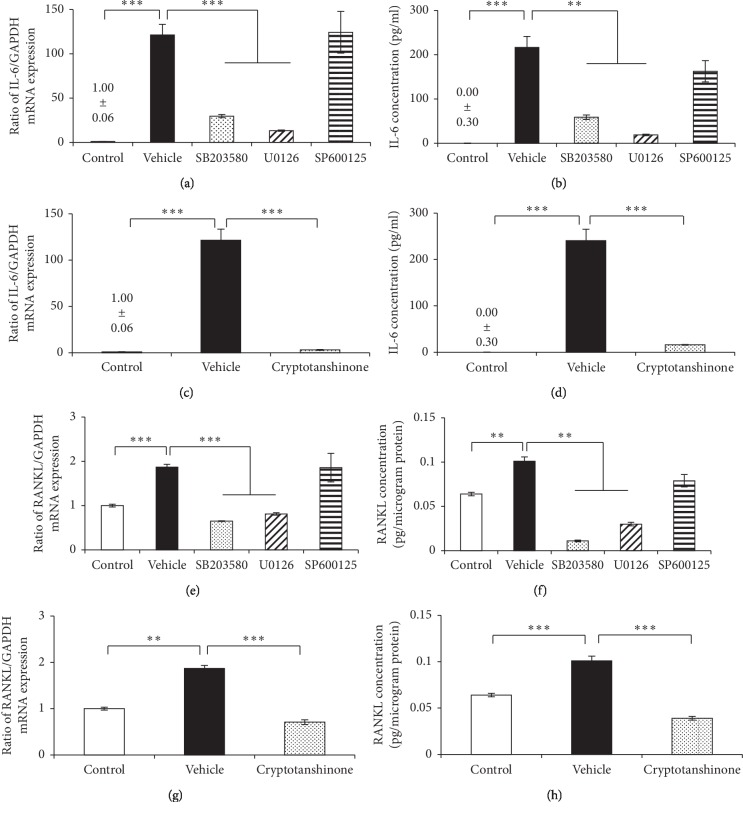
The effect of MAPKs and STAT3 inhibitors on S100A9-induced IL-6 and RANKL expressions in osteocytes. MLO-Y4-A2 cells were pretreated with SB203580 (p38 inhibitor, 20 *μ*M), U0126 (ERK inhibitor, 10 *μ*M), SP600125 (JNK inhibitor, 10 *μ*M), or Cryptotanshinone (STAT3 inhibitor, 10 *μ*M) for 1 hour, and treated with S100A9 (50 nM) for 24–48 hours. ((a), (c), (e), and (g)) Total RNA was isolated at 24 hours, and mRNA expressions were analyzed by qRT-PCR. ((b), (d), (f) and (h)) Supernatant and cell lysate were collected at 48 hours, and protein levels of IL-6 and RANKL were quantified by ELISA. All results of qRT-PCR were normalized by GAPDH mRNA expression levels. Data are means ± SD (*n* = 3). ^*∗∗*^*P* < 0.01 and ^*∗∗∗*^*P* < 0.001 show significant differences between the indicated groups.

To investigate the signaling pathways underlying S100A9-induced IL-6 and RANKL expressions, osteocytes were treated with MAPK or STAT3 inhibitor for 1 hour before the stimulation with S100A9. SB203580 (p38 inhibitor) and U0126 (ERK inhibitors) significantly downregulated S100A9-induced IL-6 and RANKL at both mRNA and protein levels. However, SP600125 (JNK inhibitor) did not affect IL-6 and RANKL at both mRNA and protein levels (Figures 7(a), 7(b), 7(e), and 7(f)). Moreover, S100A9-induced IL-6 and RANKL expressions were decreased by the treatment with cryptotanshinone (STAT3 inhibitor) (Figures 7(c), 7(d), 7(g), and 7(h)).

## 4. Discussion

Calprotectin, a heterodimer of S100A8 and S100A9, is increased in various inflammatory conditions and classified as DAMPs and alarmin because of its important roles in regulating inflammatory responses [26]. Release of calprotectin from cells is associated with infection, cellular stress, tissue damage, and cancer [27]. Periodontitis is a chronic inflammatory disease mainly caused by infection of oral microorganisms such as *Pg*, *Tannerella forsythia* and *Treponema denticola* and leads to the destruction of periodontal tissues, such as alveolar bone supporting teeth [28]. In patients with periodontitis, the plasma concentration of calprotectin is significantly higher than in healthy patients [4]. The present study showed that both S100A8 and S100A9 do not increase IL-8 and TNF-*α* mRNA expressions, and IL-1*β* is not detected in osteocytes (Figure 2). In contrast, S100A9, but not S100A8, increased both IL-6 and RANKL mRNA expressions in osteocytes and also increased IL-6 and RANKL protein levels in osteocytes by approximately 80- and 4-fold compared with control, respectively (Figure 3). Although IL-1 and TNF-*α* inhibit the differentiation of osteoblasts through MAPK signaling and sclerostin expression in osteocytes [29, 30], both S100A8 and S100A9 did not change the expressions of these proinflammatory factors in this study. On the other hand, IL-6 has important roles in both bone resorption and bone formation in healthy and disease conditions. In the initial stage of inflammation, it has been reported that IL-6 is produced from various cell types, and LPS increased the expression of IL-6 in osteocyte-like cells MLO-Y4 [31]. We also demonstrated similar results showing *Pg* LPS (as positive control) significantly increased IL-6 mRNA and protein expressions (Figure 3). IL-6 forms the complex by binding to the ligand specific *β*-receptor subunit (IL-6R), and this complex further binds to glycoprotein 130 (gp130) in bone-constructing cells, such as osteoblasts, osteoclasts, and osteocytes [32]. Kaneshiro et al. reported that the complex of IL-6 and soluble form of IL-6R (sIL-6R) decreased alkaline phosphatase activity, mRNA expressions of Runx2, osterix and osteocalcin, and bone nodule formation in osteoblasts [33]. Furthermore, it has been reported that RANKL expression was upregulated by the stimulation with IL-6 and sIL-6R complex in MLO-Y4 cells [16]. On the other hand, sIL-6R is found in serum [34], and it is conceivable that S100A9-induced IL-6 binds sIL-6R in FBS and then upregulates RANKL production. These findings suggest that calprotectin directly or indirectly affects bone metabolism through IL-6 and RANKL expressions.

In general, S100A8 and S100A9 are usually secreted at similar rates as calprotectin. Wu and Davidson reported that the different expression patterns between S100A8 and S100A9 in incisional wounds of mouse skin were not clearly observed [35]. In addition, serum levels of calprotectin were approximately 2-fold higher in patients with aggressive periodontitis than in healthy patients [36], possibly suggesting that calprotectin in serum can easily affect osteocytes in bone tissues. However, the effects of S100A8 and S100A9 on functions of osteocytes have not been fully elucidated yet. In our present results, only S100A9, but not S100A8, increased IL-6 and RANKL expressions in osteocytes (Figure 3). Previous studies have demonstrated various effects of S100A9 on inflammation in a variety of cells, growth of cancer cells and activation of natural killer (NK) cells. Gao et al. reported that S100A9 increased IL-6 and IL-8 productions in human periodontal ligament cells via TLR4 signaling pathway [37]. Furthermore, S100A9 increased the productions of IL-1*β*, IL-6, TNF-*α*, and monocyte chemoattractant protein (MCP)-1 via TLR4 pathway in human gingival fibroblasts, although induction levels of these proinflammatory mediators by stimulation with S100A8 at 50 nM were very little compared with S100A9 [38]. On the other hand, downregulation of S100A9 inhibited cellular proliferation, migration, and tumor formation through inactivating MAPKs and nuclear factor-kappa B (NF-*κ*B) signalings in human osteosarcoma cells [39]. Arnold et al. demonstrated that human immunodeficiency virus (HIV)-1-infected human monocyte-derived dendritic cells induces the modulation of S100A9 expression, potentially implying the influences of anti-HIV-1 activity of human NK cells [40]. Moreover, it has been previously reported that S100A8 and S100A9 have dependent or independent functions, and these functions could be regulated at least in part by different mechanisms [41]. These reports and our present results suggest that S100A8 and S100A9 have different functions and S100A9 possibly has significant catabolic effects on the expression of inflammatory- and bone metabolism-related factors.

It is well recognized that calprotectin activates MAPKs and NF-*κ*B signalings after binding to RAGE and TLR4. S100A9 interacts with RAGE and promotes cell growth of human hepatocellular carcinoma cells by activating ERK1/2 and p38 MAPK signaling pathways [42]. In other reports, S100A8/A9 increased the phosphorylation of p38 MAPK, and the productions of TLR4 and cyclooxygenase-2 in human aortic endothelial cells [43]. Furthermore, previous several reports demonstrated that S100A8 and S100A9 activate NF-*κ*B which mediates important functions in cellular interaction, cell survival and differentiation, and expressions of cytokines and chemokines [44]. RAGE and TLR4 were constitutively expressed in osteocytes at high level, but these expressions were not changed by the stimulation with S100A9 (Figure 4). Importantly, we found that RANKL expression in osteocytes was suppressed into similar levels to control group by the downregulation of RAGE and TLR4 using siRNA transfection (Figure 5). These results showed that S100A9 directly increased IL-6 expressions through activation of RAGE and TLR4 signaling pathways without upregulation of these receptor expressions and implied that RANKL expression levels might be strongly inhibited by the downregulation of IL-6 through RAGE and TLR4 signaling pathways. In addition, we demonstrated that S100A9 enhanced the phosphorylation of p38 and ERK1/2 MAPKs and STAT3, but not JNK (Figure 6), and the expressions of IL-6 and RANKL in S100A9-stimulated osteocytes were downregulated by the treatment with SB203580 (p38 inhibitor), U0126 (ERK inhibitor), or cryptotanshinone (STAT3 inhibitor) (Figure 7). Advanced glycation end-products (AGEs), which is one of the main ligand of RAGE, also increase IL-6 and RAGE productions and activate MAPK and NF-*κ*B in MLO-Y4-A2 cells [45]. Furthermore, LPS from *Escherichia coli* increased TLR4 expression levels in human outgrowth endothelial cells, and TLR4 signaling activates two different kinases like phosphatidylinositol-4,5-biphosphate 3 kinases (PI3K) and MAPK, as well as NF-*κ*B [46, 47]. Regarding the relatively late activation of STAT3 after the stimulation with S100A9, it is still unclear whether STAT3 activation is involved via S100A9-RAGE and -TLR4 signaling pathways, but it is conceivable that the complex of S100A9-induced IL-6 and its soluble receptor, sIL-6R, in serum can possibly activate STAT3. Moreover, the recent study also reported that pretreating MLO-Y4 cells with IL-6 increased the secretion of RANKL via the phosphorylation of STAT3 and Janus activated kinase (JAK)2, and suggested that the upregulation of RANKL affected via IL-6-JAK2-STAT3 pathway leads to osteocyte-mediated osteoclastogenesis [16]. Preliminary experiments regarding the crosstalk between MAPKs and STAT3 signaling pathways are currently under investigation to clarify this detailed mechanism of the *in vivo* mouse model as well as the *in vitro* cell culture system.

In conclusion, we demonstrated that S100A9, one component of calprotectin, activates p38, ERK, and STAT3 signaling pathways, resulting in increased IL-6 and RANKL expressions in mouse osteocyte-like cells. S100A9-RAGE and TLR4 signalings have not been fully elucidated yet in this study, and further investigations are required to clarify the signaling pathways involved in S100A9-induced IL-6 and RANKL expressions with regard to bone metabolism and inflammation. Finally, we suggest that S100A9-RAGE and -TLR4 pathways in osteocytes may be potential targets for the therapy against bone destruction mediated by the host immune and inflammatory responses to the microbial challenge, such as periodontitis.

## 5. Conclusion

In conclusion, our study showed that S100A9, not S100A8, activates ERK and p38 MAPKs, and STAT3 signaling pathways via engagement with RAGE and TLR4 to increase IL-6 and RANKL expressions in osteocytes and suggested that S100A9 may play important roles in bone destruction through the regulation of IL-6 and RANKL expressions mediated by the host immune and inflammatory responses. Furthermore, these findings may also lead to the development of new therapeutic strategies and treatments targeted on S100A9 for periodontal alveolar bone destruction.

## Figures and Tables

**Table 1:** Sequences of mouse primers for PCR.

Gene	Primer	Sequence
IL-1*β*	Forward	5′-TTCCAGGATGAGGACATGAGC-3′
	Reverse	5′-GTGCAGTTGTCTAATGGGAACG-3′

IL-6	Forward	5′-GAGGATACCACTCCCAACAGACC-3′
	Reverse	5′-AAGTGCATCATCGTTGTTCATACA-3′

IL-8	Forward	5′-AGAGCTTGAGTGTGACGCC-3′
	Reverse	5′-CCAGGTCAGTTAGCCTTGCC-3′

TNF-*α*	Forward	5′-ACTGAACTTCGGGGTGATCG-3′
	Reverse	5′-GCTACAGGCTTGTCACTCGAA-3′

RANKL	Forward	5′-TGATGAAAGGAGGGAGCACG-3′
	Reverse	5′-GATCCAGCAGGGAAGGGTTG-3′

RAGE	Forward	5′-AGGAACGTGCAGAGCTGAAT-3′
	Reverse	5′-CTGGTTGGAGAAGGAAGTGC-3′

TLR4	Forward	5′-GGCAACTTGGACCTGAGGAG-3′
	Reverse	5′-GCTAGCAGCCATGTGTTCC-3′

GAPDH	Forward	5′-GTGTTCCTACCCCCAATGTG-3′
	Reverse	5′-AGGAGACAACCTGGTCCTCA-3′

## Data Availability

The data used to support the findings of this study are included within the article.
